# Interactions between Innate Lymphoid Cells and Cells of the Innate and Adaptive Immune System

**DOI:** 10.3389/fimmu.2017.01422

**Published:** 2017-10-30

**Authors:** Cornelia Symowski, David Voehringer

**Affiliations:** ^1^Department of Infection Biology, University Hospital Erlangen and Friedrich-Alexander University Erlangen-Nuremberg (FAU), Erlangen, Germany

**Keywords:** allergic inflammation, helminths, mast cells, innate lymphoid cells, eosinophils, basophils, Th2 cells, alternatively activated macrophages

## Abstract

Type 2 innate lymphoid cells (ILC2s) are a major source of cytokines, which are also produced by Th2 cells and several cell types of the innate immune system. Work over the past few years indicates that ILC2s play a central role in regulating type 2 immune responses against allergens and helminths. ILC2s can interact with a variety of cells types of the innate and adaptive immune system by cell–cell contacts or by communication *via* soluble factors. In this review, we provide an overview about recent advances in our understanding how ILC2s orchestrate type 2 immune responses with focus on direct interactions between ILC2s and other cells of the immune system.

## Introduction

Innate lymphoid cells (ILCs) are characterized by their lack of expression of rearranged antigen-receptors and absence of cell surface markers present on other common hematopoietic cell lineages. They arise from a common lymphoid progenitor, requiring expression of the transcriptional repressor Id2 and relying on cytokine signaling through the common gamma-chain (γ_c_ chain) of the IL-2 receptor family. ILCs are classified into three distinct populations termed group 1, 2, and 3 ILCs based on their ability to produce effector cytokines associated with Th1, Th2, or Th17 cells, respectively (1–4).

Group 2 ILCs (ILC2s) share the expression of the transcription factor GATA-3 and characteristic cytokines with Th2 cells indicating a similar role for both cell types in type 2 immune responses (5, 6). ILC2s respond extremely rapidly to epithelial cell-derived cytokines, such as IL-25, IL-33, and thymic stromal lymphopoietin (TSLP), associated with barrier disruption (7) and act as “early sentinel” cells of the innate immune system orchestrating type 2 immune responses at mucosal surfaces and adipose tissue (8–10).

The canonical type 2 immunity-associated cytokines IL-4, IL-5, and IL-13 are pivotal in immunity toward gastrointestinal helminths inducing eosinophilia, elevated IgE levels, goblet cell metaplasia with enhanced mucus production, and smooth muscle hyperreactivity (11, 12). It is evident that cells of the adaptive and innate immune system, including Th2 cells, ILC2s, eosinophils, basophils, and mast cells, produce significant levels of these cytokines inducing and sustaining ongoing type 2 responses (13, 14). Although the prominent role of Th2 cells in type 2 immune responses is well established, the secretion of IL-13 from ILC2s rather than Th2 cells is of particular importance for controlling the intestinal immune response and worm expulsion during infection of mice with the helminth *Nippostrongylus brasiliensis* (9, 10, 15). On the other hand, T cell-derived IL-4/IL-13 are needed for effector cell recruitment, germinal center formation, IgE switching, and paracrine Th2 differentiation (16, 17).

In contrast to the protective function of ILC2s, dysregulated ILC2 responses contribute to inflammatory processes, such as airway hyperreactivity (18), allergen-induced lung inflammation (19, 20), and atopic dermatitis (21). Despite the substantial gain of knowledge about ILC2s development and mediators that positively or negatively modulate ILC2 homeostasis, activation, and functions (22, 23), the regulation of ILC2 functions is becoming more complex, and it is of high importance to understand the immunoregulatory mechanisms to improve therapeutic treatments of pathological type 2 immune responses. Besides producing cytokines, ILC2s may interact with other effector immune cells and coordinate immune responses as part of the complex immune system network important for immune defense and allergic reactions. Recent data indicate that ILC2s can influence T cell responses in a reciprocal manner, either through cytokines, indirect effects on accessory cells, or direct cell–cell contact relaying signals to the adaptive immune system. Additionally, ILC2s also contribute to the maintenance of eosinophils (24) and affect the functions of cells such as basophils (25), macrophages (26), dendritic cells (DCs) (27, 28), and mast cells (29), which on the other hand can also activate ILC2s (30) or suppress their activity (31).

Defining the complex network of interactions and mutual communications of ILC2s with immune cells from the innate and adaptive immune system and understanding the specific contributions of ILC2s leading to protective immunity against helminths or development of pathologic responses may reveal critical checkpoints that can be manipulated for controlling type 2 immunity-mediated responses and will be important to investigate new possible therapeutic interventions.

## Interactions of ILC2s with Cells of the Adaptive Immune System

### ILC2s and T Cells

Th2 cells are a major source of IL-4 and IL-13 and they play an important role in type 2 immune responses. Recently, our group revealed that specific depletion of IL-4/IL-13 in CD4^+^ T cells results in reduced accumulation of innate effector cells (eosinophils, basophils, ILC2s) in the lung of *N. brasiliensis*-infected mice as compared to wild-type mice and that CD4^+^ T cell-derived IL-4/IL-13 cytokines promote Th2 polarization in a paracrine manner (16, 17). Beside eosinophils, basophils, and mast cells, mouse ILC2s are also known to transcribe and produce low amounts of IL-4 induced by the lipid mediator leukotriene D4 (15, 32, 33). Leukotrienes can act in a synergistic manner together with IL-33 to activate ILC2s (34, 35). Human ILC2s appear to secrete larger quantities of IL-4 as compared to mouse ILC2s especially upon IL-33 stimulation and in the presence of TSLP (36). During infection with the helminth *Heligmosomoides polygyrus*, ILC2-derived IL-4 has been shown to contribute to ILC2 expansion and to drive early Th2 differentiation without direct cell–cell contact (33). Furthermore, mice lacking ILC2s showed impaired Th2-skewed inflammatory responses following helminth infection or local exposure to the protease-allergen papain, or house dust mite antigens (27, 37, 38). This suggests that ILC2s can promote Th2 responses under certain experimental conditions. In addition, IL-13-producing ILC2s were shown to cooperate with CD4^+^ T cells during *N. brasiliensis* infection to mediate larval killing in the small intestine during primary infection (38) and in the lung following secondary infection (26). Furthermore, *N. brasiliensis* could be expelled by transfer of ILC2s into IL-13-deficient mice, but not into Rag2-deficient mice (9). This indicates that IL-13 from ILC2s is sufficient for clearance of primary *N. brasiliensis* infection, but CD4^+^ T cells are still required for effective worm expulsion Interestingly, T cell-derived IL-2 can induce ILC2 proliferation and IL-13 secretion (39). In addition, it was shown that in mice exposed to the pro-allergic protease papain ILC2-derived IL-13 rather than IL-4 promotes migration of DCs into lung-draining lymph nodes, where activated DCs support Th2 cell differentiation (27).

Innate lymphoid cells not only contribute to Th2 cell differentiation by cytokine secretion but can also directly interact with CD4^+^ T cells. Using an *in vitro* culture system, it was reported that ILC2s promote Th2 polarization in a cell–cell contact-dependent manner (39). In addition, both costimulation by OX40/OX40-L interaction and ILC2-derived IL-4 was shown to enhance Th2 cell proliferation and Th2 cytokine production when the isolated cell populations were cultured together (40). Beside expressing costimulatory molecules, ILC2s have also been shown to express MHC class II (9, 39, 41). Recent data identified ILC2s as antigen-presenting cells (APC) able to process and present peptide antigens and modulate naive CD4^+^ T cell activation in a cell contact-dependent manner (38, 39, 42). Expression of MHC-II on ILC2s was required to receive activating signals by T cell-derived IL-2 causing efficient secretion of IL-13 (38). This suggests that ILC2s and T cells can communicate in an antigen-dependent manner. However, whether ILC2s play a significant role as APC during priming of the Th2 response remains to be investigated.

### ILC2s and Treg Cells

Subsequent studies demonstrated that Treg cells and ILC2s engage in reciprocal regulation. Treg cells are regulators of adaptive immune responses through direct cell–cell contact, as well as through the suppressive activities of IL-10 and TGF-β. The importance of Treg cells on control of ILC2 activity and homeostasis has recently been shown by inhibition of the transcription factors Id2 and Id3 in Treg cells, which lead to a spontaneous increase in ILC2 counts, as well as accumulation of eosinophils in the lungs and resulted in the development of fatal inflammatory disease (43). While Treg cells regulate ILC2 expansion and suppress their pro-inflammatory cytokine secretion *in vivo* and *in vitro*, ILC2-derived IL-4 plays a requisite role in mediating sensitization to food allergens by compromising allergen-specific regulatory T cell function and thereby promoting food allergy (44). In contrast, ILC2-derived IL-9 was required for Treg activation and resolution of inflammation in an arthritis model (45). In addition, ILC2s may enhance the suppressive activity of Treg cells by secretion of amphiregulin as it has been described for mast cell-derived amphiregulin (46).

IL-33 can either directly activate ST2^+^ Treg cells in the intestine (47) or promote their expansion indirectly by inducing IL-2 secretion from DCs (48). Others have shown that accumulation of Treg cells in adipose tissue by IL-33 was directly dependent on ILC2s by a process requiring ILC2-intrinsic MyD88 signaling and cognate interactions between inducible T cell co-stimulator (ICOS) on Treg and ICOS ligand (ICOS-L) on ILC2s (49). As IL-33 alone can activate significant Treg cell proliferation independently of ILC2s, one can assume that ILC2s act mainly to promote Treg survival. Coculture experiments revealed that ICOS-L/ICOS interaction is important for the ILC2-mediated survival of Treg cells (49). Furthermore, ILC2-derived IL-4 may promote the conversion of Treg cells to Th2 cells after *H. polygyrus* infection (50). A recent study demonstrated that induced Treg cells (iTreg), but not natural Treg cells, effectively suppressed human and mouse ILC2 function and this effect was dependent on ICOS/ICOS-L interactions (51). Autocrine ICOS/ICOS-L interactions on ILC2s were also reported to play an important role to enhance survival of ILC2s (42, 52). IFN-γ was found to counter-regulate the effects of IL-33 mediated ILC2 activation and Treg cell expansion (49), indicating that Th1-dominated immune responses can actively suppress the IL-33-ILC2-Treg axis and thereby cause loss of barrier tissue integrity and shifts in fat metabolism. Understanding the contributions of ILC2s and Tregs to optimal immune regulation might help preventing excessive tissue damage and the development of chronic inflammations.

### ILC2s and B Cells

In addition to their mutual interactions with T cells, ILC2s may also interact with B cells. It is known that fat-associated lymphoid clusters (FALCs), found in human and in mouse mesentery, contain large proportion of B1 cells and contribute to innate B cell activation and germinal center differentiation (53). So far, it is described that ILC2s from FALCs maintain homeostasis of the self-renewing B1 cells in an IL-5-dependent manner and support production of parasite-specific antibodies by B cells (8, 54). Interestingly, it was described that lung ILC2s not only enhance the proliferation of B1 but also follicular B2 type B cells and promote the secretion of IgM, IgG1, IgA, and IgE by these cells in the absence of T cells (55). It is also assumed that ILC2s have the ability to promote T cell-dependent antibody responses as serum IgE levels are reduced in ILC2-deficient mice challenged with papain (27). Human ILC2s were found to directly activate B cells isolated from tonsils (56).

Furthermore, it has been described that ILC2s can enhance IgE production from B cells *in vitro*, raising the possibility that ILC2s orchestrate IgE-dependent allergic sensitization (57). Whether ILC2s can interact with ICOS-L-expressing B cells is still an open question and additional studies are required to identify B cell-derived mediators that feedback on ILC2s. However, the addition of anti-ICOS antibodies showed no effect on the ability of ILC2s to enhance B cell IgM and IgG1 production indicating that cytokines like IL-5 and other soluble factors that are secreted by ILC2s may play a key role (55).

**Figure 1:**
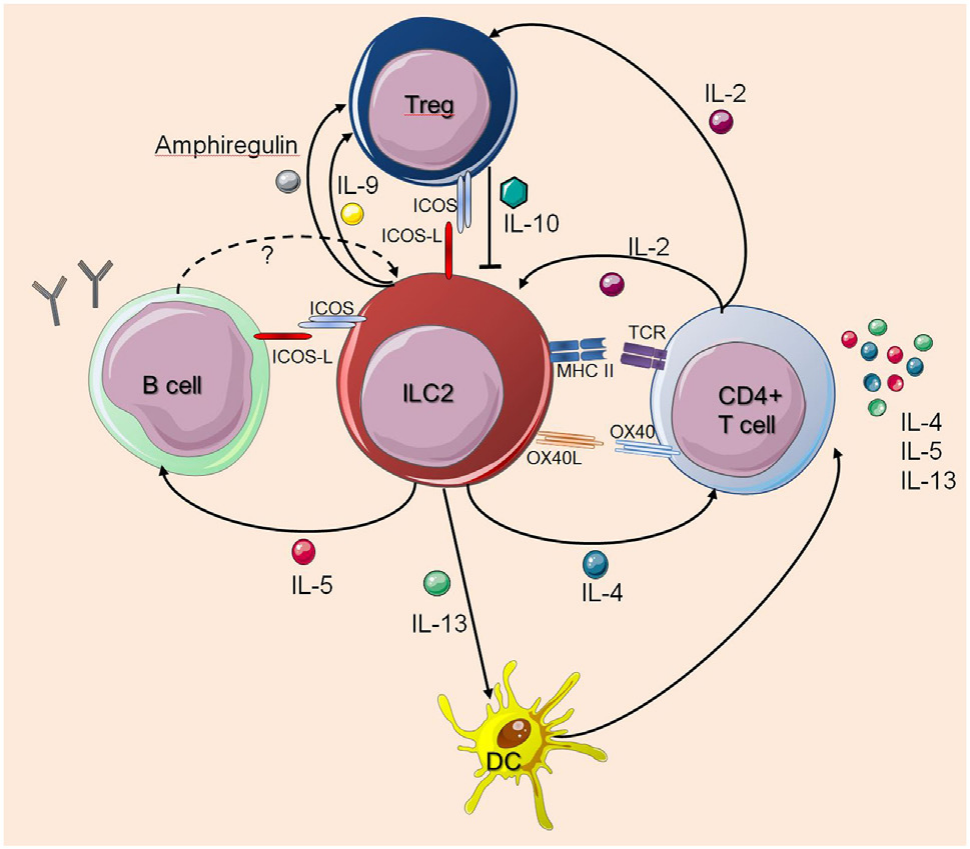
Interactions between innate lymphoid cells (ILC2s) and T cells or B cells. The figure illustrates how ILC2s interact with T cells or B cells as described in the main text. The dashed arrow indicates a potential feedback regulation for which there is currently no experimental evidence. Solid arrows indicate published evidence for activating mechanisms whereas Treg-derived IL-10 was shown to suppress ILC2s. Cell symbols where taken from http://smart.servier.com website.

Still, many questions remain to be answered regarding the interactions between ILC2s and cells of the adaptive immune system. Taken together, ILC2s regulate and dictate the nature of downstream T cell- and B cell-mediated immune responses, while T cells also influence the survival, proliferation, and function of ILC2s (Figure 1).

## Interactions of ILC2s with Cells of the Innate Immune System

The alarmins IL-25, IL-33, and TSLP initiate a local inflammatory response through the recruitment and activation of ILC2s and other innate effector cells. Investigating the role of ILC2s during coordination of this early innate immune response is critical to uncover their contribution for type 2 immunity during infections, allergic responses, or autoimmune diseases.

### ILC2s and Macrophages

Innate lymphoid cells can promote the differentiation of so-called “alternatively activated” or M2 macrophages, which have protective functions in some helminth infection models and contribute to tissue repair responses (58). Using a mouse model for cerebral malaria, IL-33-elicited ILC2s were found to promote M2 polarization and Treg cell expansion resulting in protective immunity (59). It was further described that IL-33- or IL-2-elicited ILC2s are sufficient to induce M2 macrophage-mediated larval killing of *N. brasiliensis* helminths in the lung (26). Antifungal type 2 immune responses are also regulated by ILC2-mediated M2 polarization as demonstrated with a *Cryptococcus neoformans* infection model (60). In addition, IL-25-elicited ILC2-derived IL-13 was shown to promote activation of lung-resident macrophages to a profibrotic phenotype, driving collagen deposition from fibroblasts (61).

M2 macrophages in visceral adipose tissue (VAT) play an important role for glucose and fat metabolism and ILC2s as well as eosinophils have been described to promote M2 macrophage accumulation in VAT during helminth infection (62, 63). Furthermore, it has been reported that IL-33-activated ILC2s elicit the differentiation of alternatively activated macrophages through IL-4 receptor signaling and regulate directly beige fat biogenesis (64). However, recent studies indicate that IL-4-mediated macrophages have no relevant effect on white and brown adipocyte function and do not likely modulate adipocyte metabolism by catecholamine production (65, 66).

The other way round, alveolar macrophages are able to secrete IL-33, which is likely important for direct activation of ILC2s to produce substantial amounts of IL-13, as mice lacking the IL-33 receptor failed to develop AHR or airway inflammation independently of adaptive immunity (67). Thus, cross talk between macrophages and ILC2s might be critical to promote an early feed forward process during type 2 immune responses.

### ILC2s and DCs

Type 2 innate lymphoid cell (ILC2)-derived IL-13 was reported to induce migration of DCs into the draining lymph node, where DCs drive naive T cells to become Th2 cells (27). However, how IL-13 controls the migratory function of DCs still remains to be defined. Furthermore, ILC2-derived IL-13 promotes the secretion of the chemokine CCL17 from DCs for the recruitment of CCR4^+^ memory Th2 cells to the site of allergen exposure (28). Eosinophils recruited into tissues and lymph nodes can also control DC activation and migration and promote Th2-cell-mediated immunity *via* degranulation of eosinophil peroxidase (68), demonstrating how innate cells work in a complex network to drive type 2 inflammatory responses.

The tumor necrosis factor (TNF) family cytokine TL1A is known to be produced by DCs and macrophages in response to toll-like-receptor and Fc receptor cross-linking and regulates the adaptive immune response by co-stimulating T cells (69). It was demonstrated that TL1A synergizes with IL-25 *in vivo* to directly promote ILC2 expansion, survival, and function (70, 71). The activation of ILC2s by TL1A could provide new insight into interaction of ILC2s and activated myeloid cells.

Recent observations revealed that type I and type II IFNs as well as IL-27 play a critical role as negative regulators of ILC2s to restrict type 2 immunity and its associated pathologies (49, 72–74). It has been reported that plasmacytoid dendritic cells (pDC) play a critical role in dampening the function and survival of ILC2s in the context of allergic pulmonary inflammation. IFN-α production by activated pDCs can inhibit proliferation and increases the apoptosis rate of ILC2s (31). Similarly, polyinosinic–polycytidylic acid (pI:C) activated NK cells inhibit the proliferation and cytokine production of ILC2s *via* IFN-γ during the early stage of lung inflammation, reminescent of the classic antagonism between Th1 and Th2 differentiation (75). As interferons are produced by many different cell types including Th1 cells, NKT cells, and others, one can assume that ILC2s may be suppressed by various cellular sources of these mediators.

### ILC2s and Eosinophils

Innate lymphoid cells are a major source of IL-5 and thereby enhance proliferation, survival, and recruitment of eosinophils (24, 49). By comparing Rag2^−/−^ and Rag2^−/−^ γ_c_^−/−^ mice, lung ILC2s were shown to promote eosinophilia independently of signals from the adaptive immune system (76). Naive lung ILC2s secrete IL-5 constitutively, as indicated by elevated *Il5* mRNA levels and fluorescent signals in *Il5*-reporter mice (24, 77). ILC2-derived IL-5 is required for systemic maintenance of eosinophils and during type 2 inflammation ILC2s are induced to co-express IL-13, resulting in localized eotaxin production for recruitment and activation of eosinophils during allergic inflammation and helminth infection (24, 62, 78). It was further shown that vasoactive intestinal peptide, a hormone regulated by caloric intake, stimulates ILC2s *via* the vasoactive intestinal peptide receptor 2 receptor to release IL-5, linking eosinophil levels with metabolic cycling (24). IL-33 can directly stimulate eosinophil survival (79) and activate the production of IL-4 (80), which can stimulate ILC2s and thereby mediate the cross talk between eosinophils and IL-5-producing ILC2s (81).

### ILC2s and Basophils

Basophils were shown to promote ILC2 proliferation by secretion of IL-4 in lung and skin inflammation models (25, 82). Basophils are relatively short-lived circulating cells, which have specified effector functions in type 2 immunity such as protective functions against helminths and ticks as well as pro-inflammatory functions in response to allergens. Lung ILC2 activation and numbers are reduced in basophil-specific IL-4-deficient mice, indicating that the ILC2s respond to basophil-produced IL-4 (25, 83). Furthermore, clusters of basophils and ILC2s where shown to accumulate in a mouse model for atopic dermatitis where basophil-derived IL-4 was required for ILC2 accumulation and proliferation in inflamed skin (82). Whether basophil activity, survival, or tissue recruitment is regulated by ILC2-derived factors remains to be analyzed.

### ILC2s and Mast Cells

Innate lymphoid cells have been found in proximity to tissue mast cells in human lung (84). Furthermore, dermal ILC2s and skin-resident mast cells have been reported to physically interact in contact dermatitis models in mice using intravital multiphoton microscopy, supporting the idea that ILC2s can directly communicate with mast cells (85). It was shown that ILC2s have the potential to dampen pro-inflammatory mast cell response through the production of IL-13, thereby reducing IL-6 and TNF-α production by mast cells (85).

The close proximity of ILC2s and mast cells could be caused by production of inflammatory mediators such as PGD2 by mast cells, which induces the chemotaxis of ILC2s through activation of cysteine-three-histidine protein 2 and promotes IL-13 production in a synergistic manner with airway epithelial cytokines IL-25 and IL-33 leading to tissue eosinophilia (84, 86–88). Moreover, mast cells can influence ILC2 activity indirectly by releasing non-caspase proteases chymase and tryptase that cleave IL-33 into a more bioactive isoform (89).

Mast cells can also release IL-33 upon antigen-specific IgE-mediated activation (90) and in response to extracellular ATP, which in turn activates ILC2s to produce IL-13 resulting in clearance of helminth infection (30). Conversely, recombinant IL-33 can directly activate mast cells to produce several cytokines including IL-4 and IL-5. IL-33-activated mast cells are known to produce IL-2, which was shown to indirectly limit ILC2 proliferation by the expansion of Treg cells and their production of IL-10 (91). On the other hand, mast cells also produce IL-2 when activated by IL-9 from IL-33-elicited ILC2s. This mast cell-derived IL-2 leads to expansion of pro-inflammatory CD25^+^ ILC2s, which in turn activate Th9 cells leading to an amplified allergic inflammation (92). However, comparing *N. brasiliensis*-infected wild-type mice with IL-9 receptor-deficient mice showed similar mast cell accumulation in the lung arguing against a major role of ILC2-derived IL-9 for mastocytosis in this model (93).

## Conclusion

**Figure 2:**
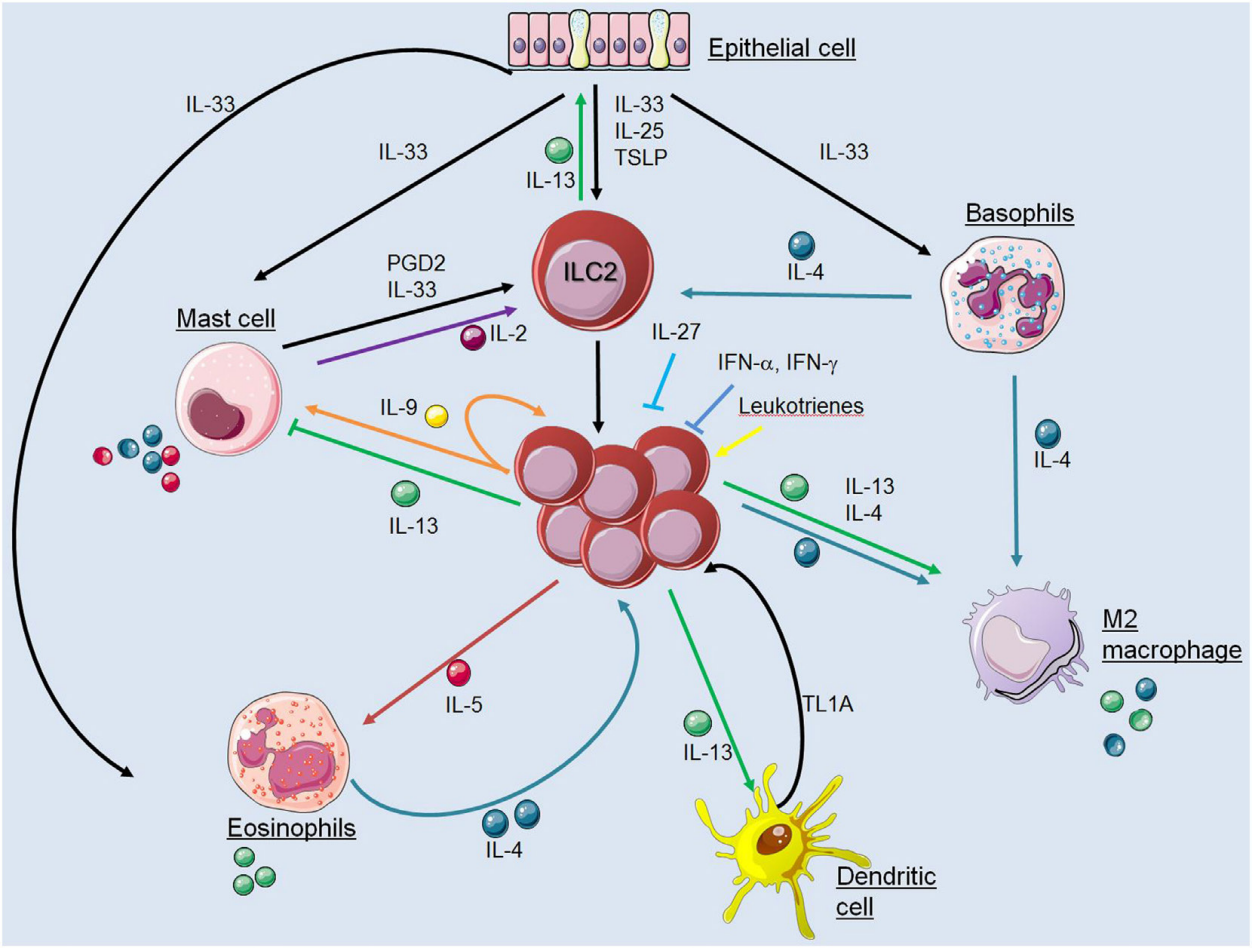
Interactions between innate lymphoid cells (ILC2s) and cells of the innate immune system. The figure illustrates communication pathways between ILC2s and other cells of the innate immune system by secretion and recognition of soluble factors as described in the main text. Solid arrows indicate published evidence for activating mechanisms whereas ILC2-derived IL-13 was shown to suppress mast cells and IL-27 or interferons inhibit ILC2 functions. Cell symbols where taken from http://smart.servier.com website.

Innate lymphoid cells are important “early sentinel” cells, which bridge the gap between the innate and adaptive type 2 immune response by sensing environmental changes and releasing immune-regulatory cytokines. A large variety of pathways that regulate the functions of ILC2s have been identified in the recent past and key interactions between ILC2s and cells of the innate and adaptive immune system were characterized (Figure 2). Apart from cross talk with various immune cell types, ILC2s may also have effects on structural cells including epithelial cells, smooth muscle cells, and fibroblasts. These interactions in mice and humans include communications by direct cell–cell contact and by secretion and recognition of soluble factors like cytokines, chemokines, hormones, and lipid mediators. However, regulation of ILC2 functions in various tissues during steady state conditions and upon infection with different pathogens becomes more and more complex and many pathways are still unknown. Further investigations will help to improve our understanding of how interactions between ILC2s and other immune cells are regulated. This information is essential to dissect the complexity of type 2 immune responses with the hope to identify critical checkpoints that are accessible for therapeutic interventions in allergic inflammation and immunity against helminths.

## Author Contributions

All authors listed have made a substantial, direct, and intellectual contribution to the work and approved it for publication.

## Conflict of Interest Statement

The authors declare that the research was conducted in the absence of any commercial or financial relationships that could be construed as a potential conflict of interest.

## Abbreviations

ILC, innate lymphoid cells; PGD2, prostaglandin 2; CRTH2, cysteine-three-histidine protein 2; NK cell, natural killer cell; VAT, visceral adipose tissue; CLP, common lymphoid progenitor; ICOS, inducible T cell co-stimulator; VPAC2, vasoactive intestinal peptide receptor 2; FALC, fat-associated lymphoid cluster.
